# The evolution, variation, and expression patterns under development and stress responses of the NAC gene family in the barley pan-genome

**DOI:** 10.3389/fpls.2025.1635416

**Published:** 2025-08-07

**Authors:** Xin Liu, Minghu Zhang, Jian Su, Lei Wu, Mang Shen, Yamei Zhuang, Qi Wang, Gang Chen

**Affiliations:** ^1^ Faculty of Agriculture, Forestry and Food Engineering, Yibin University, Yibin, Sichuan, China; ^2^ Crop Research Institute, Shandong Academy of Agricultural Sciences, Jinan, Shandong, China; ^3^ State Key Laboratory of Crop Gene Exploration and Utilization in Southwest China, Sichuan Agricultural University, Chengdu, Sichuan, China; ^4^ Key Laboratory of Aquatic Genomics, Ministry of Agriculture and Rural Affairs, Beijing Key Laboratory of Fishery Biotechnology, Chinese Academy of Fishery Sciences, Beijing, China

**Keywords:** Hordeum vulgare, NAC, pangenome, evolutionary dynamics, pantranscriptome, salt stress response

## Abstract

The NAC transcription factor family is pivotal in regulating plant development and stress responses, yet its diversity and evolutionary dynamics in barley (*Hordeum vulgare*) remain underexplored. In this study, we performed a comprehensive pan-genome analysis to identify and characterize the *HvNACs* across 20 barley accessions. A ranging from 127 to 149 *HvNACs* were identified in each genome, in which the Morex genome harbored the highest count. These *HvNACs* were classified into 201 orthogroups, further stratified into core (102), soft-core (18), shell (25), and lineage-specific (56) categories. Phylogenetic analysis delineated them into 12 subfamilies, of which the core genes have undergone strong purifying selection, by contrast, the shell and lineage-specific genes were under relaxed selection constraint, suggesting functional diversification in barley. Genomic variation, such as PAVs and CNVs, largely driven by TEs, highlighted the dynamic nature of NAC loci. Furthermore, transcriptome profiling of the *HvNACs* demonstrated diverse tissue expression patterns and different response characteristics under salt stress. These findings elucidate the evolutionary and functional dynamics of *HvNACs*, offering valuable insights for genetic improvement of breeding programs in barley as well as in other crops.

## Introduction

NAC transcription factors, emerged during the transition from aquatic to terrestrial plant life and expanded thereafter, establishing themselves as one of the largest transcription factor families in plants (Mathew and Agarwal, 2018; Chen and Xia, 2025). They modulate an array of biological processes, such as secondary growth, cell division, senescence, hormone signaling, and nutrient uptake (Xiong et al., 2025). Moreover, NAC transcription factors coordinate multiple developmental and environmental signals, creating complex regulatory networks that precisely adjust target gene expression (Li et al., 2011; Nuruzzaman et al., 2013; Parankusam et al., 2017). These proteins possess a conserved N-terminal NAC domain (~150 amino acids) and a highly variable C-terminal transcriptional regulation region, which exhibit functional specificity through their structural diversity The NAC domain consists of five subdomains (designated A to E), each performing distinct molecular functions (Mohanta et al., 2020). Of these, subdomains C and D are highly conserved and essential for DNA binding, while subdomain A supports homo- and heterodimerization, enabling interactions with other NAC proteins. By contrast, the more variable subdomains B and E promote the functional diversification of NAC proteins across plant species (Kim et al., 2016). The C-terminal region shows considerable sequence variation and functions as a transcriptional activation or repression domain, often interacting with other regulatory proteins to modulate gene expression (Hu et al., 2010; Mohanta et al., 2020). This dual-domain structure supports the diverse roles of NAC transcription factors in regulating essential physiological processes and aiding stress adaptation.

NAC transcription factors serve as pivotal regulators of plant responses to both biotic and abiotic stresses (Sun et al., 2012; Shao et al., 2015). These plant-specific transcription factors feature conserved DNA-binding domains and function as either activators or repressors in a variety of cellular processes (Singh et al., 2021). They engage in intricate signaling networks and transcriptional reprogramming crucial for stress adaptation (Nuruzzaman et al., 2013). Notably, NAC transcription factors often exhibit auto-regulation and cross-regulation, enabling them to coordinate responses to multiple stresses simultaneously (Yuan et al., 2019). For example, *ANAC019*, *ANAC055*, and *ANAC072/RD26* have been demonstrated to enhance drought tolerance in *Arabidopsis thaliana* (Tran et al., 2004). The *ANAC072/RD26* functions as transcription activator in ABA-dependent stress-signaling pathway (Fujita et al., 2004). In rice (*Oryza sativa*), the stress-responsive *SNAC3* boosts heat and drought tolerance by regulating reactive oxygen species homeostasis (Fang et al., 2015). Moreover, *ANAC096* and bZIP transcription factors work together to mediate dehydration and osmotic stress responses in *Arabidopsis* (Xu et al., 2013). Overexpressing of grapevine *VvNAC08* and wheat (*Triticum aestivum*) *TaNAC29* has also been shown to enhance drought and salt tolerance in transgenic *Arabidopsis* (Xu et al., 2013). In addition to abiotic stress tolerance, NAC transcription factors play vital roles in plant immunity. In wheat, the NAC protein *GRAB1–2* binds to the wheat dwarf geminivirus RepA protein, regulating viral DNA replication (Xie et al., 1999). Considering the increasing environmental challenges in modern agriculture, the NAC transcription factors offer promising avenues for improving crop resilience. Overexpressing stress-responsive NAC genes has been successfully used in transgenic approaches to improved tolerance to various stresses (Nuruzzaman et al., 2013). However, precise promoter selection and rigorous field validation are essential to mitigate potential trade-offs between stress tolerance and plant growth (Shao et al., 2015). Future research should aim to unravel the intricate regulatory mechanisms of NAC transcription factors and refine their application in breeding stress-resilient crop varieties.

Genome-wide studies have identified a large number of NAC genes across diverse plant species, such as there are 105 NAC genes in *Arabidopsis* (Ooka et al., 2003), 154 in tobacco (*Nicotiana tabacum*) (Li et al., 2018), 151 in rice(*Oryza sativa*) (Nuruzzaman et al., 2010), 559 in wheat (Guerin et al., 2019), and 147 in foxtail (*Setaria italica*) (Puranik et al., 2013). Furthermore, a comparative genomic analysis of 24 land plant species revealed a total of 3,187 NAC transcription factors, which were clustered into six major groups. These findings highlight the evolutionary complexity of the NAC gene family, marked by variations in gene number and phylogenetic relationships across species. Traditional gene family identification often relies on identifying conserved domains within a single reference genome. However, this approach fails to capture the full extent of genetic diversity within species, particularly gene presence/absence variation (gPAV), which is common among individuals (Tettelin et al., 2005). To gain a more comprehensive understanding of species-wide diversity in genome, the concept of the pan-genome has emerged and gained significant attention. Unlike traditional approaches that rely on a single reference genome, pangenome overlook gene presence/absence variation among individuals, which integrates genomic data from multiple accessions, reconstructing the species’ complete gene repertoire (Jayakodi et al., 2021). There are two categories for genes included in a pangenome, the core genes shared across all individuals and the dispensable/variable genes present only in subsets of accessions (Bayer et al., 2020). Pan-genome analysis provides a robust framework for exploring intra-species genetic variation, adaptive evolution, domestication, and functional diversity (Tong et al., 2025).

Barley (*Hordeum vulgare*), one of the world’s oldest cultivated cereal crops, ranks the fourth-largest crop after maize, rice, and wheat in terms of yields. It possesses broad adaptability to diverse environments and plays essential roles in feeding, malting, and brewing. Cultivated barley (*H*. *vulgare* subsp. *vulgare*) was domesticated approximately 10,000 years ago from its immediate ancestor wild barley (*H. vulgare* subsp. *spontaneum*) (Zohary et al., 2012), which exhibits substantial genetic diversity potentially improving barley cultivars. Barley has been well developed in genetics, genomics, and breeding over the past two decades, particularly in pan-genomics in recent five years. The pan-genome assembly consisting of 19 cultivated accessions and one wild barley accession has been published (Jayakodi et al., 2020), and subsequently released a barley pan-transcriptomic datasets of 20 barley genotypes (Guo et al., 2025). The published barley pan-genome have facilitated functional genomics analyses, several studies have performed a pan-genomic approach for gene-family characterization and exploring structural variations during domestication process in barley (Chen et al., 2022; Jayakodi et al., 2024; Tong et al., 2025). In this study, we systematically identified the NAC gene family members in barley at the pan-genome level. We further analyzed gene structures, physicochemical properties, chromosomal localization, phylogenetic relationships, and tissue-specific expression patterns. These approaches addressed the limitations of single-reference genome studies and provided a more comprehensive understanding of the *HvNACs*’s evolutionary dynamics and functional diversity in barley. Moreover, we explored the potential roles of *HvNACs* in salt stress responses, offering new insights into stress tolerance mechanisms and a theoretical basis for genetic breeding improvement in barley.

## Materials and methods

### Identification of *HvNACs*

The genomic sequences of 20 barley pangenome accessions were retrieved from the IPK database (https://barley-pangenome.ipk-gatersleben.de) as described by Jayakodi et al. (2020). The genome and protein sequence data of rice (Oryza sativa. IRGSP-1.0) were downloaded from the Ensembl database (http://asia.ensembl.org/index.html). The NAM domain (PF02365) hidden Markov model (HMM) file was downloaded from the Pfam website (https://pfam.xfam.org/). Using HMMER (v3.0) software with an E-value threshold of 1E-5 and a minimum domain score of 20, as described by Chen et al. (2022), HvNAC protein sequences containing the NAM domain were identified. To further confirm NAC domain presence, BlastP (v2.9.0) was used with an E-value threshold of 1E-10 and a minimum query coverage of 70% against the Pfam NAM domain sequence. These potential protein sequences were further validated for the presence of the NAC domain using the NCBI Conserved Domain Database (CDD; https://www.ncbi.nlm.nih.gov/cdd/), the SMART database (http://smart.embl-heidelberg.de/), and the Pfam database.

Orthologous *HvNACs* clusters across the 20 barley pangenome accessions were inferred using the CD-HIT (v4.6) (Li and Godzik, 2006) with sequence identity threshold of 95% and alignment coverage of 90% for the longer sequence, requiring a minimum alignment length of 100 amino acids. Orthologous gene groups (OGGs) were defined as clusters of NAC genes sharing at least 95% sequence identity and were further classified based on their presence across accessions: core OGGs (present in all 20 accessions), soft-core OGGs (present in 18–19 accessions), shell OGGs (present in 3–17 accessions), and cloud OGGs (present in only one accession). To calculate copy number variation (CNV), we counted the number of gene members in each OGG for every barley accession. The resulting OGG-by-accession matrix was used to compare gene copy numbers across the 20 genomes. The identified pangenome *HvNACs* were sequentially numbered and sorted according to their conservation category and the order of their corresponding OGGs. The longest protein in each cluster was designated as the representative sequence. The physicochemical properties of the NAC proteins were computed using the ExPASy online tool (https://web.expasy.org/protparam/). To visualize the presence/absence patterns of NAC genes across the 20 barley genomes, we used the R package ComplexHeatmap (Gu et al., 2016) to generate a heatmap. Additionally, ggplot2 (Villanueva and Chen, 2019) was used to create plots summarizing the frequency distribution of NAC gene occurrence across accessions.

### Phylogenetic tree construction

The phylogenetic tree of NAC proteins was constructed using the Maximum Likelihood (ML) method implemented in IQ-TREE v2.0 (Nguyen et al., 2015). Full-length amino acid sequences of NAC proteins from barley and rice were first aligned using MUSLE. The resulting multiple sequence alignment was then trimmed using trimAl with the automated option to remove poorly aligned regions and reduce noise. The best-fit substitution model (Dayhoff) was selected automatically by IQ-TREE. To assess the robustness of phylogenetic relationships, bootstrap analysis was performed with 1,000 replicates. Based on the clustering results of the phylogenetic tree and previously reported classifications in rice and miaze (Ooka et al., 2003; Peng et al., 2015), the NAC proteins were assigned to distinct subfamilies. The final phylogenetic tree (*.nwk file) was uploaded to the iTOL platform (http://itol.embl.de/) for enhanced visualization and further annotation.

### Analysis of gene structures and conserved motifs

Intron-exon structures for representative *HvNACs* were obtained from the annotation files corresponding to 20 barley genomes. Additionally, conserved motifs within the *HvNACs* of barley were identified utilizing the MEME Suite (http://meme-suite.org/meme), specifying a maximum limit of 10 motifs. Subsequently, the resulting gene structures and motif distributions were visualized employing TBtools (v2.097) (Chen et al., 2020).

### Analysis of duplication events and collinearity

Synteny analysis for *HvNACs* was conducted using MCScanX (Wang et al., 2013), utilizing amino acid sequences and corresponding chromosomal positions from the Morex V3 genome assembly (https://wheat.pw.usda.gov/GG3/) (Mascher et al., 2021) These syntenic relationships were subsequently visualized using a circos plot generated with TBtools (v2.097) (Chen et al., 2020). To further explore evolutionary selection pressures, synonymous (Ks) and non-synonymous (Ka) substitution rates were calculated for orthologous gene pairs across 20 barley accessions. This estimation utilized the approximate method available in KaKs Calculator (v1.2) (Zhang et al., 2006). A Ka/Ks ratio greater than 1 indicates positive selection, a ratio equal to 1 suggests neutral selection, and a ratio less than 1 signifies purifying selection.

### Analyses of presence/absence variation and transposable element

Datasets for PAV and TE covering the Morex genome and 19 additional barley genomes were obtained from the source indicated by https://doi.org/10.5447/ipk/2020/24. Subsequently, custom Python scripts were employed to identify TE regions overlapping *HvNACs*. These overlapping TE elements were then categorized based on their position relative to *HvNACs* as upstream, downstream, or genic. Furthermore, the transposons themselves were classified according to the three-letter code system outlined by Wicker et al. (2007).

### Codon usage evaluation

Codon usage bias was analyzed using codonW (v1.4.2) (http://codonw.sourceforge.net/). The following parameters were calculated: effective number of codons (ENC), codon adaptation index (CAI), relative synonymous codon usage (RSCU), overall genomic G+C content (GC), G+C content at the third synonymous codon position (GC3s), and nucleotide composition at the third synonymous codon position (T3s, C3s, A3s, G3s). Optimal codons were identified based on genome-wide ENC values. Genes in the lowest and highest 10% ENC categories were classified as high-expression and low-expression gene sets, respectively. Optimal codons were defined as those with an RSCU > 1 in high-expression genes, RSCU < 1 in low-expression genes, and a ΔRSCU (RSCU difference) ≥ 0.3.

To assess the relative contributions of natural selection and mutational pressure on codon usage bias, Parity Rule 2 (PR2) analysis was performed. PR2 plots were generated by calculating the A3/(A3 + T3) ratio and G3/(G3 + C3) ratio at the third codon position for each HvNAC across the 20 barley accessions. A reference point at (0.5, 0.5) was included to indicate a state of equal mutational pressure and no bias between purines and pyrimidines. ENC-plot analysis was conducted to evaluate codon usage patterns and the effect of GC content at the third synonymous codon position (GC3s). ENC values were plotted against GC3s for each NAC gene, and the expected ENC values under a neutral mutation model were computed using the formula:


$$ENC\;=\;2\;+\;GC3s\;+\;\frac{29}{\left[\text{GC}3{\text{s}}^{2}\;+\;(1-\text{GC}3{\text{s}}^{2})\right]}$$

Genes with ENC values falling below this curve were inferred to be under selection rather than mutational pressure. All plots, including PR2 and ENC analyses, were visualized using Matplotlib (v3.5.2) (Barrett et al., 2005), with consistent color coding across barley accessions to facilitate comparative analysis.

### Transcriptome analysis

Raw transcriptome sequencing data from five distinct tissues (caryopsis, coleoptile, inflorescence, root, and shoot) across 20 barley genomes were utilized in this study, sourced from a recent comprehensive barley transcriptome investigation (Guo et al., 2025). Initially, raw sequencing reads underwent processing with Trimmomatic (v0.39) (Bolger et al., 2014) to eliminate adapter sequences, low-quality reads, and potential contaminants, thereby ensuring high data integrity. Subsequently, the filtered reads were aligned to the Morex V3 reference genome assembly (https://wheat.pw.usda.gov/GG3/) (Mascher et al., 2021) using HISAT2 (v2.2.1) (Kim et al., 2019), noted for its efficient and accurate mapping capabilities. Transcript assembly and quantification were then performed using StringTie (v2.1.3) (Pertea et al., 2015) based on the alignment results. Gene expression variation across the different tissues was assessed by calculating the average FPKM value for each *HvNAC* across all 20 barley genomes. Finally, these expression patterns were visualized as a heatmap generated with the ComplexHeatmap package in R (v4.4.0) (Gu et al., 2016), facilitating the analysis of tissue-specific expression profiles and potential functional divergence among the *HvNACs*.

### Analysis of gene expression clustering

To investigate the salt stress response of *HvNACs* in barley, transcriptome data of leaf tissues subjected to varying durations of salt treatment (0 h, 1 h, 24 h, and 10 d) were retrieved from the SRA database (accession ID: PRJNA962512). In this experiment, barley seedlings were cultivated under controlled conditions until the three-leaf stage, at which point they were treated with 300 mM NaCl to induce salt stress. Leaf samples were collected at the designated time points following treatment for RNA sequencing and subsequent expression analysis. Expression patterns across these time points were analyzed using the Mfuzz package (Kumar and Futschik, 2007), as previously described by Chen et al. (2023), resulting in the classification of genes into 10 distinct clusters based on their temporal expression profiles. The fuzzification parameter (m) was optimized at 1.25 to balance cluster membership stringency and flexibility. To explore the co-expression relationships, Pearson correlation analysis was performed to calculate the correlation between *HvNACs* in each cluster and other genes, with a threshold of correlation coefficient greater than 0.9 and p-value less than 0.01 applied to identify highly correlated genes. Additionally, NAC transcription factor binding motifs were downloaded from the PlantTFDB database (https://planttfdb.gao-lab.org/prediction.php). The FIMO (v5.3.3) (Grant et al., 2011) was employed to search for these motifs within the promoter sequences of genes highly correlated with *HvNACs*, enabling the identification of potential regulatory targets. Visualization of the co-expression networks and motif analysis results was performed using Cytoscape (v3.10.2) (Su et al., 2014). Gene Ontology (GO) enrichment analysis was subsequently performed to elucidate the potential functional roles associated with these modules. Gene Ontology terms for the enrichment analysis were retrieved from the KOBAS database (http://bioinfo.org/kobas). Visualization of the results was performed using relevant R packages to facilitate a comprehensive understanding of the molecular mechanisms governing the salt stress response of *HvNACs*.

## Results

### Identification of *HvNACs* in 20 barley pangenome

Using HMMER and BlastP, we identified *HvNACs* across 20 barley genomes. To eliminate redundancy and facilitate downstream analyses, we used CD-HIT to cluster similar sequences. For each cluster, one representative gene per accession was retained. The number of *HvNACs* in each genome ranged from 127 (HOR_21599) to 149 (Morex), with most genomes (85%) containing 130–135 *HvNACs*. To further characterize these *HvNACs*, we conducted a comprehensive analysis of their physicochemical properties (**Supplementary Table S1**). The sequence lengths varied significantly, ranged from 100 amino acids to 863 amino acids, while their molecular weight spanned from 11,854.43 Da to 96,275.98 Da. The pI ranged from 4.29 to 10.57, indicating a broad spectrum of charge distributions. The instability index ranged from 27.00 to 82.12, reflecting variations in protein stability. Meanwhile, the aliphatic index spanned from 42.46 to 73.75, suggesting differences in thermostability. The GRAVY values, all negative, ranged from -0.243 to -0.951, confirming the hydrophilic nature of these *HvNACs*. The considerable diversity in physicochemical properties highlights the potential functional versatility of barley *HvNACs*.

The Morex-v3 assembly contained a significantly higher number of NAC genes than the other 19 barley genomes, prompting an investigation into whether this expansion may have resulted from improvements in genome assembly quality. To explore this, we performed a comparative analysis between Morex-v3 and its predecessor, Morex-v2, which contains 137 NAC genes. Clustering of NAC genes from both versions revealed divergences. A total of 167 clusters were identified, including 63 clusters with single genes, 103 clusters forming 1:1 pairs, and one cluster containing three genes (**Supplementary Table S2**). These results suggest differences between assembly versions, potentially including resolved fragmentation, merged loci, or novel gene annotations in v3. The analysis indicates that advancements in genome assembly continuity may contribute to more accurate identification of genes, which might help explain the elevated NAC gene count in Morex-v3 relative to both its earlier version.

### Core and dispensable *HvNACs*

**Figure 1:**
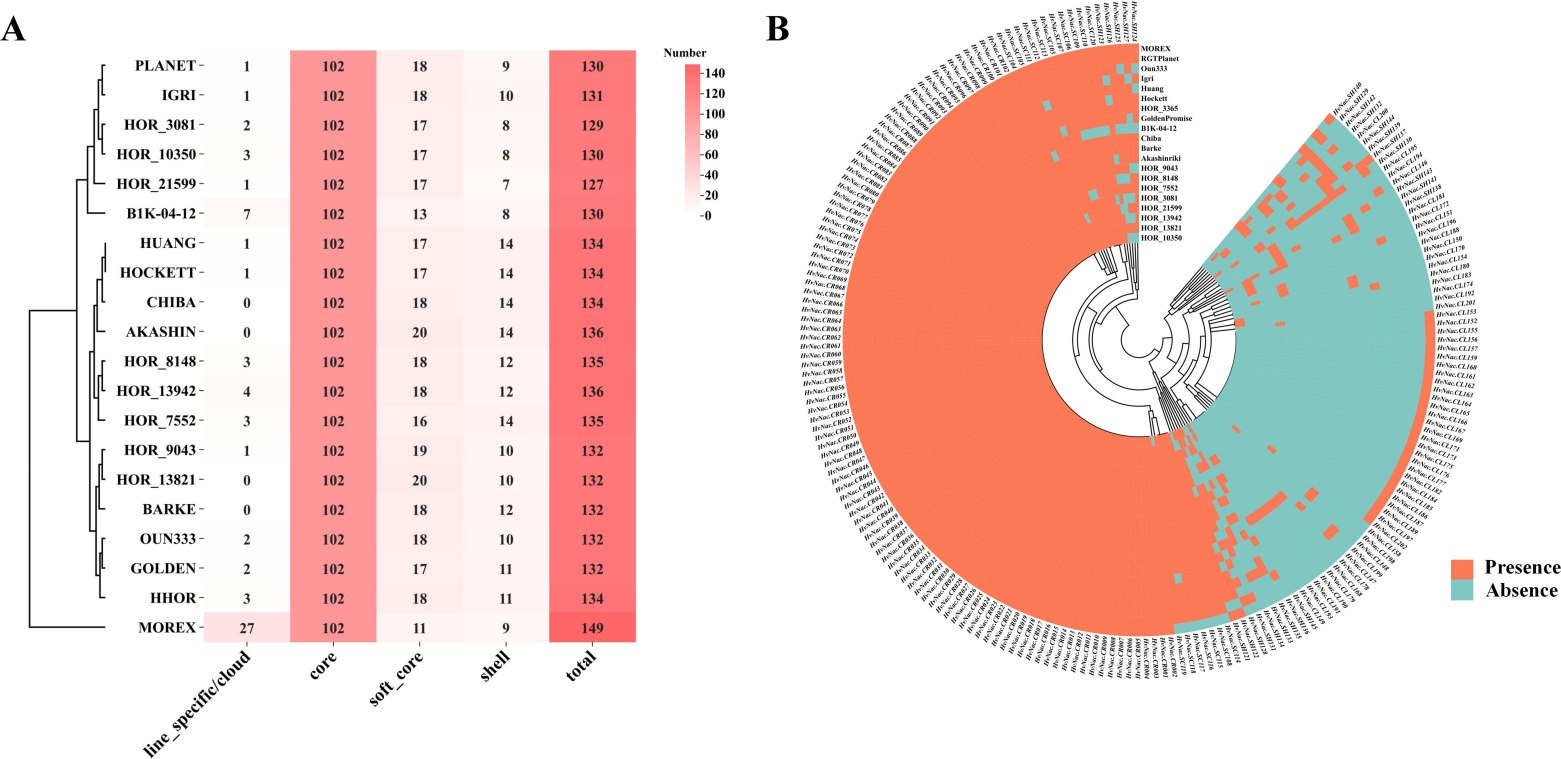
Number and distribution of *HvNACs* in the barley pangenome. **(A)** Statistics of the number of different types of NAC genes in 20 barley genomes. **(B)** Heatmap showing the presence and absence of NAC genes across 20 barley varieties. Each row represents a NAC gene, and each column represents a barley accession. Orange indicates the presence of a gene, while blue indicates its absence.

To investigate the conservation of *HvNACs* in the barley pan-genome, we assigned the *HvNACs* identified from 20 barley accessions into different orthologous gene groups (OGGs). Using a threshold of ≥95% amino acid sequence identity, a total of 2,842 *HvNACs* from the 20 barley genomes were clustered into 201 orthologous gene groups (OGGs).The 201 OGGs were stratified into four conservation categories (**Supplementary Table S3**; **Figures 1A, B**): (1) Core clusters (n=102), exhibiting strict evolutionary conservation with all 20 genes present in every accession, likely maintaining essential cellular functions; (2) Soft-core clusters (n=18), displaying near-ubiquitous distribution (conserved in ≥90% lines) with limited lineage-specific losses; (3) Shell clusters (n=25), showing moderate conservation (present in >10% but <90% of the accessions) and missing in 3–17 genomes, indicative of evolutionary plasticity; and (4) Lineage-specific/cloud clusters (n=56), characterized by extreme diversification (≤10%) and absence in 18–19 accessions, potentially encoding adaptive traits. This conservation-to-divergence continuum not only delineates fundamental versus specialized NAC functions but also provides an evolutionary framework prioritizing candidate genes during barley improvement.

### Phylogenetic analysis of *HvNACs*

**Figure 2:**
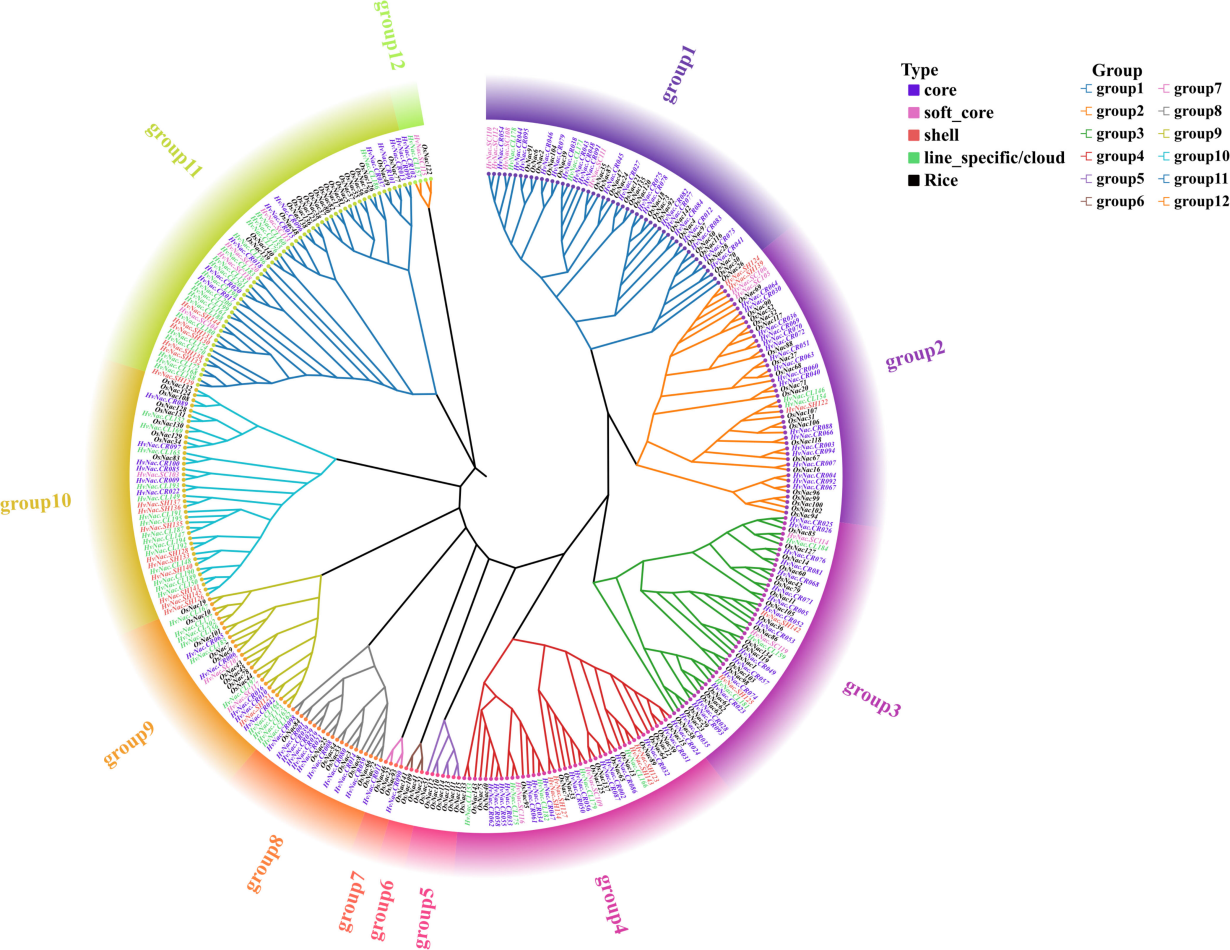
Phylogenetic tree of barley NAC genes. The evolutionary tree was constructed using IQ-TREE with the maximum likelihood method (1,000 bootstrap replicates) based on the Dayhoff model.

Phylogenetic analysis of *HvNACs* in the barley pan-genome and rice (based on the *Oryza sativa* reference genome, IRGSP-1.0) identified 12 major subfamilies (group 1 to group 12), with groups 5 and 6 comprising only rice NAC genes, suggesting potential lineage-specific functional divergence (**Supplementary Table S3**; **Figure 2**). In barley, core and soft-core genes, collectively accounting for 81.2% of all NAC entries, were predominantly clustered in group 1 (23.7%), group 2 (15.3%), group 3 (19.5%), group 4 (14.4%), and group 8 (8.3%), suggesting conserved evolutionary roles in essential regulatory processes. By contrast, lineage-specific/cloud and shell genes, which comprised 18.8% of total entries, were enriched in groups 9 to 12. Notably, group 10 (7.1%) and group 11 (6.5%) contained the highest proportions of dynamic, species-specific genes, likely contributing to adaptive diversification. Interestingly, group 7 harbored only a single HvNAC gene. The absence of *HvNACs* in groups 5 and 6, along with their exclusive presence in rice, underscores the divergent evolutionary trajectories of these two Poaceae species. This pattern illustrates that conserved subfamilies retain essential functions, reflecting a balance between genetic stability and evolutionary innovation within the NAC gene family.

### Analysis of gene structure and conserved motif

To investigate the evolutionary relationships and structural diversity of *HvNACs* within the barley pangenome, we performed a comprehensive analysis of their gene structures and conserved protein motifs (**Supplementary Table S4**). Our analysis reveals that *HvNACs* within the same subfamily typically display consistent exon-intron architectures and motif compositions, highlighting their close evolutionary relationships. Most *HvNACs* contain 1 to 6 exons. Motif analysis identified a conserved core set of motifs (Motif_0 to Motif_6) shared by most subfamilies. However, subfamily-specific deviations were evident; for example, group 10 genes (e.g., *HvNAC.CR085*) frequently lacked Motif_1 or included Motif_9, while group 11 genes (e.g., *HvNAC.CR096*) often contained Motif_7 or Motif_8, suggesting potential functional specialization. Dispensable *HvNACs* largely mirrored the motif patterns of core genes within their subfamilies; however, the presence of unique motifs in lineage-specific genes (e.g., *HvNAC.CL177* in group 1 with Motif_9) suggests adaptive divergence.

### Analysis of CNVs, PAVs, and transposon elements

**Figure 3:**
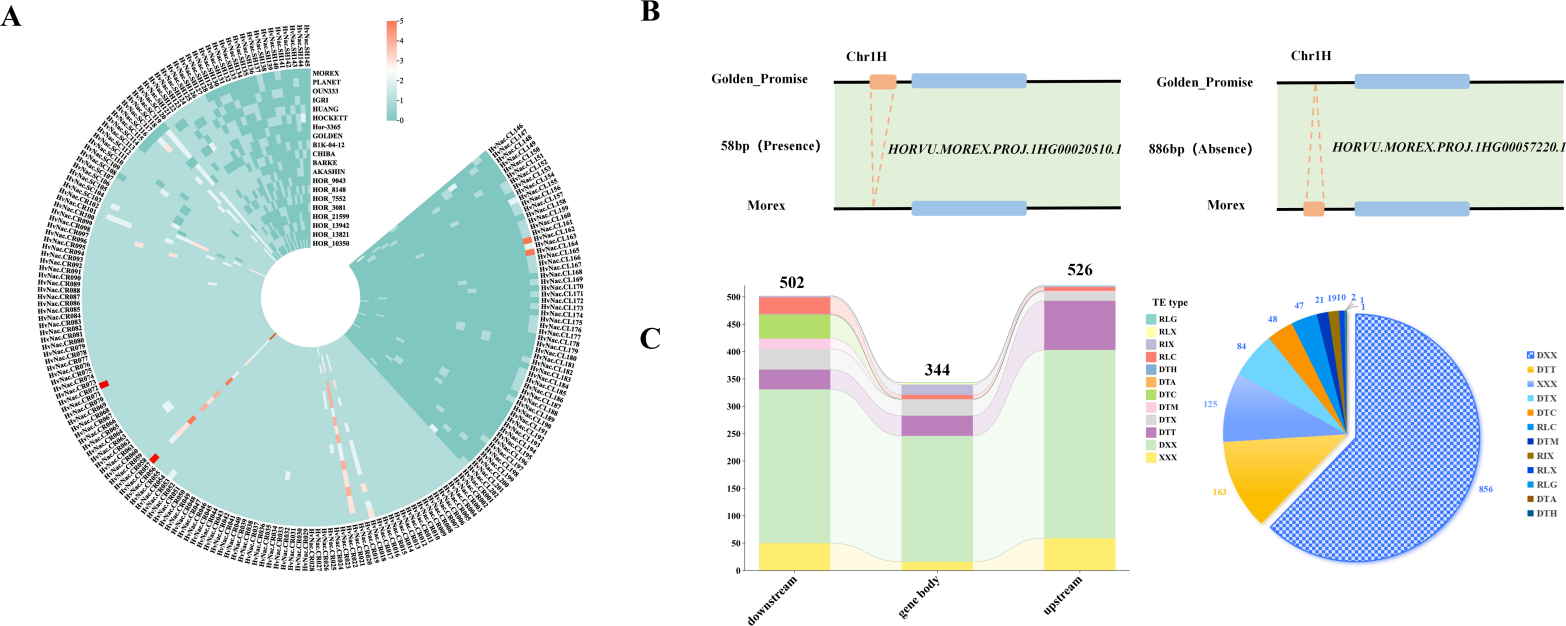
Copy number variation, presence-absence variation, and transposable element distribution of *HvNACs*. **(A)** Heatmap of NAC gene copy numbers across different barley genomes. **(B)** PAV overlapping with the NAC gene family, with two panels showing presence and absence compared to Morex. **(C)** Distribution of different types of TEs identified within 2 kb upstream and downstream of NAC genes; the left panel shows TE positions, and the right panel indicates TE types.

Analysis of CNV in *HvNACs* across the barley pangenome revealed substantial variability, with gene copy numbers ranging from 1 to 58 (**Supplementary Table S5**; **Figure 3A**). Among the 201 *HvNACs* identified, 89 were single-copy core genes consistently present across all genomes, whereas some lineage-specific genes exhibited copy number expansion in specific cultivars. For instance, genes such as *HvNAC.CR001* to *HvNAC.CR017* maintained a uniform single copy across all lines. In contrast, *HvNAC.CR021* exhibited up to four copies in cultivars including HOR_3081, HOR_7552, and Akashinriki, while *HvNAC.CR057* reached seven copies in Morex. Further analysis revealed additional complexity for CNVs of barley NAC. *HvNAC.CL162* and *HvNAC.CL164* exhibited five copies exclusively in Morex, suggesting potential cultivar-specific amplification events. This variability reflects the dynamic evolutionary of *HvNACs* and may indicate functional diversification or adaptive significance associated with specific genomic backgrounds.

PAVs analysis of NAC gene regions across the barley pan-genome, based on a comparative assessment of 19 barley genomes against the Morex reference, identified a total of 567 distinct PAV events (**Supplementary Table S6**). These included both insertions and deletions relative to the Morex genome. For example, the locus *Morex-HvNAC1* harbors a 58 bp insertion in the Golden Promise genome that is absent in Morex, whereas *Morex-HvNAC4* contains an 886 bp region present in Morex but missing in Golden Promise (**Figure 3B**). Further analysis revealed extensive PAV complexity at specific NAC loci among different barley. The *Morex-HvNAC4* locus exhibited substantial insertional variation, including a 6,822 bp segment in Barke and a 9,304 bp segment in Golden Promise, alongside deletions such as a 1,559 bp segment absent in Chiba. Similarly, the *Morex-HvNAC7* locus demonstrated remarkable PAV diversity, ranging from a 58 bp deletion in Barke to a large 553,451 bp deletion in Golden Promise, suggesting major genomic rearrangements at this locus.

TEs were identified as key contributors to NAC gene duplication in barley. A comprehensive analysis revealed 1,377 TE insertions within or flanking *HvNACs* regions across the barley pan-genome (**Supplementary Table S7**). The positional distribution of these elements showed a prevalence in potential regulatory regions: 526 (38.2%) were found upstream, 502 (36.5%) downstream of the coding sequences, and 349 (25.3%) within genic boundaries (**Figure 3C**). Notably, DNA transposons constituted the vast majority (85.25%) of these identified TEs, although various retrotransposon types, including LTR elements as Ty1-copia retrotransposons (RLC) and Ty3 retrotransposons (RLG), were also present (**Figure 3D**). In addition, NAC-associated TEs were predominantly distributed toward the distal ends of chromosomes, with chromosome 7H showing the highest overall TE abundance associated with HvNAC regions (**Supplementary Figure S1**).

### Analyses of natural selection

**Figure 4:**
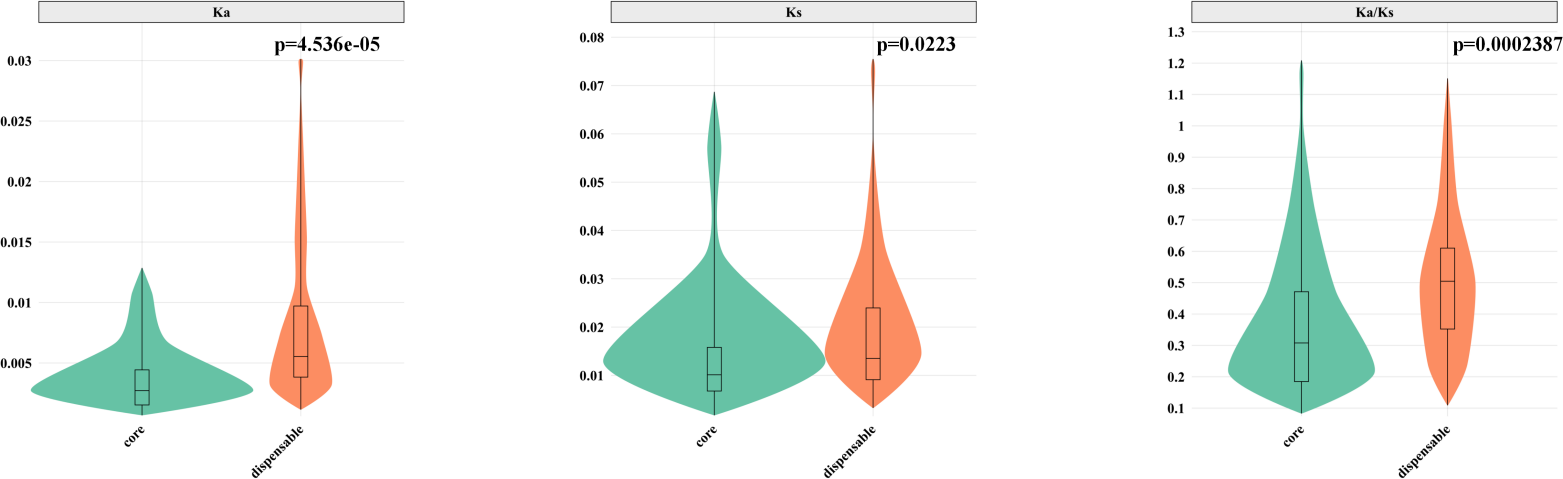
Comparison of Ka, Ks, and Ka/Ks values between core and dispensable NAC genes. P-values were calculated using the Mann-Whitney U test (non-parametric test).

In the barley pangenome, we performed an evolutionary analysis of 201 OGGs within the *HvNACs*. The results revealed that core *HvNACs* exhibited significantly lower Ka, Ks, and Ka/Ks values compared to dispensable genes (p < 0.05) (**Figure 4**), suggesting that core genes are subject to stronger purifying selection. This intensified selective constraint likely reflects their indispensable roles in essential biological processes, thereby necessitating higher sequence conservation. In contrast, dispensable *HvNACs* appear to be under relaxed selection pressure, allowing greater evolutionary plasticity and enabling the acquisition of more diverse or specialized functions.

### Collinearity analysis

**Figure 5:**
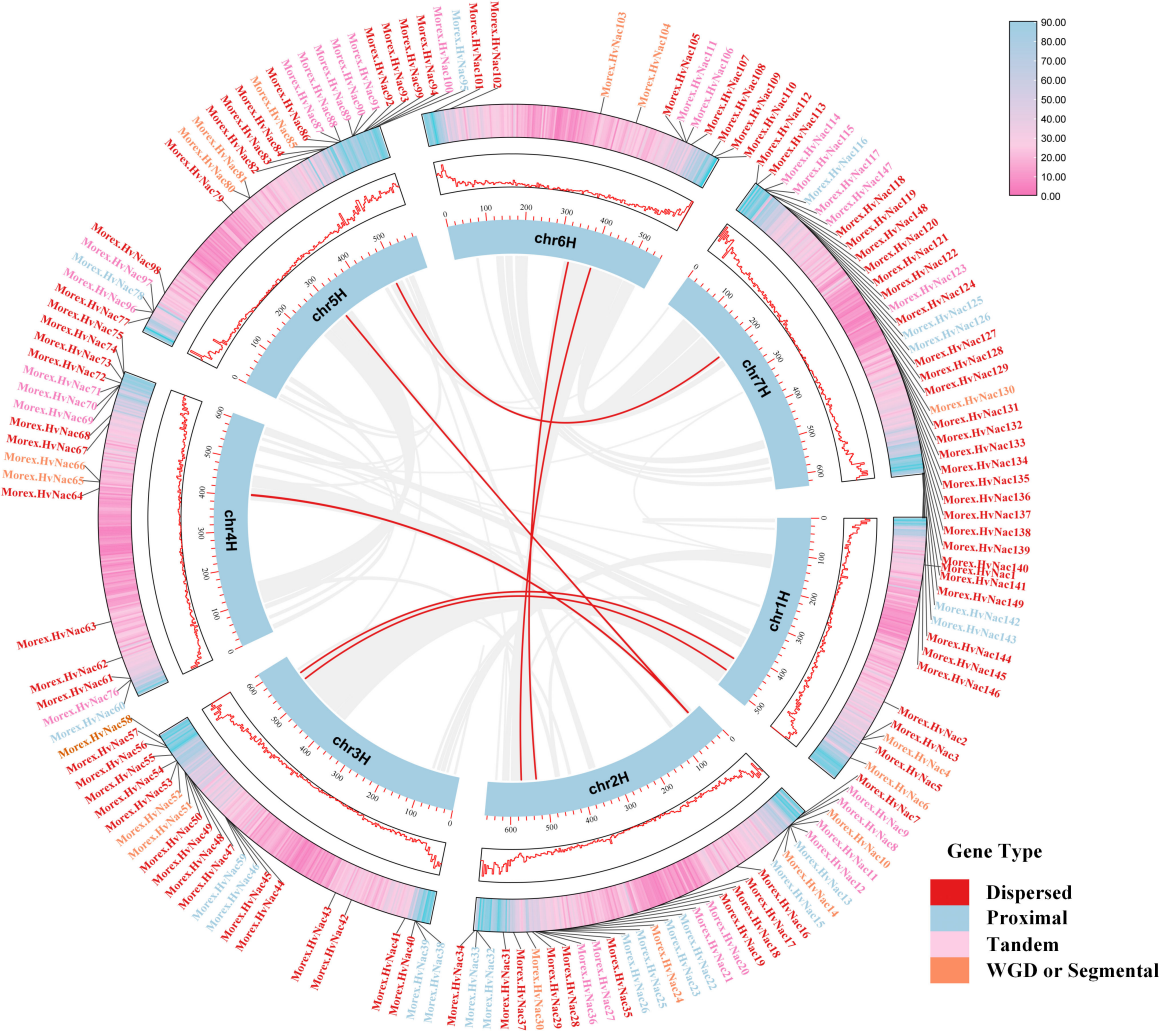
Intraspecific collinearity analysis of NAC genes in the Morex genome. Gene types are color-coded as follows: Dispersed (red), Proximal (blue), Tandem (pink), and WGD or Segmental (orange). Collinear gene pairs are connected by red lines.

In the Morex barley reference genome, the *HvNACs* displays an uneven chromosomal distribution, with notable enrichment at the distal ends of chromosomes 2H, 3H, 5H, and 7H (**Figure 5**). These telomeric and subtelomeric regions—characterized by high recombination frequencies and elevated structural variation—are recognized as hotspots for rapid gene family expansion and functional innovation. A comprehensive analysis revealed that dispersed duplication is the primary mechanism driving NAC gene expansion, accounting for approximately 56.37% of the family members (**Figure 5**). This suggests that transposon-mediated replication and large-scale segmental rearrangements have played a central role in the relocation, dispersion, and functional diversification of *HvNACs*. Additionally, tandem duplication contributes to 18.12% of NAC gene expansion, emphasizing the importance of localized gene cluster amplification, likely driven by unequal crossing-over events, increasing gene copy number and supporting functional diversity. Collectively, the observed duplication patterns and chromosomal distribution highlight a complex evolutionary history for the barley NAC gene family, shaped by diverse duplication events and selective pressures.

### Codon usage evaluation

We observed distinct codon usage patterns of NAC gene lineages across the barley pan-genome. Analyzing 2,666 *HvNACs* revealed that the effective number of codons (ENC) ranged from 26.99 to 61.00, with an average of 42.79. As most ENC values exceeded 40, this suggests a generally low codon usage bias in *HvNACs* (**Supplementary Table S8**). Furthermore, the codon adaptation index (CAI) values ranged from 0.168 to 0.356, with an average of 0.253, significantly lower than the optimal value of 1. This indicates both weak codon preference and relatively low expression potential.

The relative synonymous codon usage (RSCU) values, as presented in **Supplementary Table S9**, reveal that 29 codons had an RSCU greater than 1. RSCU is a measure of how frequently a particular codon is used to encode an amino acid, relative to the expected usage if all synonymous codons for that amino acid were used equally (Sharp et al., 1986). An RSCU value greater than 1 indicates a preference for that codon in the gene set analyzed. Among these, the preferred codons were predominantly those ending in G (13 codons) and C (16 codons). In contrast, 28 codons were underrepresented, with an average RSCU value of less than 0.6, while 13 codons were overrepresented, with an average RSCU value greater than 1.6. By comparing the RSCU values between the two codon preference libraries in *HvNACs*, we identified four optimal codons: CCC (Proline), GCT (Alanine), GAT (Aspartic acid), and CTC (Leucine) (**Supplementary Table S10**). These codons showed a ΔRSCU greater than 0.3, with RSCU values exceeding 1 in high-preference genes and less than 1 in low-preference genes.

**Figure 6:**
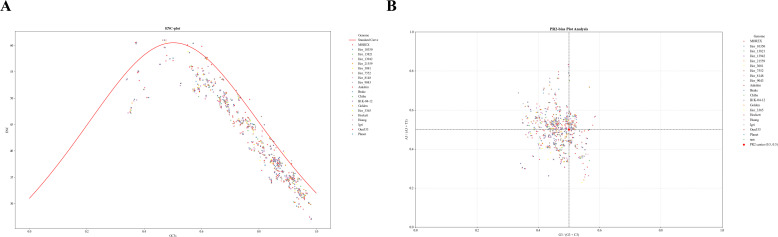
Codon usage bias analysis of NAC genes in the barley genome using ENC and PR2 plots. **(A)** ENC-plot analysis of NAC genes in the barley genome. The red line indicates the expected position of genes when codon usage is determined solely by GC3s. ENC represents the Effective Number of Codons; GC3s represents the G+C content at the third position of synonymous codons. **(B)** PR2-plot analysis of NAC genes in the barley genome. A3/(A3 + T3) represents the ratio of A to A+T at the third codon position. G3/(G3 + C3) represents the ratio of G to G+C at the third codon position.

Since ENC is influenced by the G + C content of the gene, the GC3 of the gene is commonly used to study codon usage patterns. The analysis revealed that most genes have ENC values ranging from 25 to 60, indicating varying levels of codon usage bias. The majority of data points fall below the expected curve (**Figure 6A**), suggesting that other selective forces are also shaping codon selection in these genes. The additional selective pressure is generally driven by translational selection, which favors the use of specific codons to improve translation efficiency and/or accuracy as reported by previous study (Hershberg and Petrov, 2008). Consequently, the codon usage patterns of these *HvNACs* may be shaped by both mutational bias and translational selection.

To assess codon usage bias (CUB) in the analyzed genomes, a Parity Rule 2 (PR2) plot was generated, where A_3_/(A_3_+T_3_) was plotted on the vertical axis and G_3_/(G_3_+C_3_) on the horizontal axis for each gene (**Figure 6B**). The results revealed a slight deviation of data points from the expected neutral position (0.5, 0.5), with average values of 0.4951 for A_3_/(A_3_+T_3_) and 0.4610 for G_3_/(G_3_+C_3_), suggesting a moderate codon usage bias. Most G_3_/(G_3_+C_3_) values were below 0.5, indicating a preference for cytosine over guanine at the third codon position. This bias is likely shaped by both mutational pressure and natural selection. This conclusion is further supported by the significantly higher G_3_+C_3_ content (0.768) compared to A_3_+T_3_ (0.232), reflecting a marked GC preference in the analyzed genomes. In contrast, the average A_3_/(A_3_+T_3_) value of 0.4951, with individual genes reaching as high as 0.7, suggests a relatively balanced usage of adenine and thymine, with a slight preference for adenine in specific cases.

### Expression profiling of *HvNACs* across different tissues

**Figure 7:**
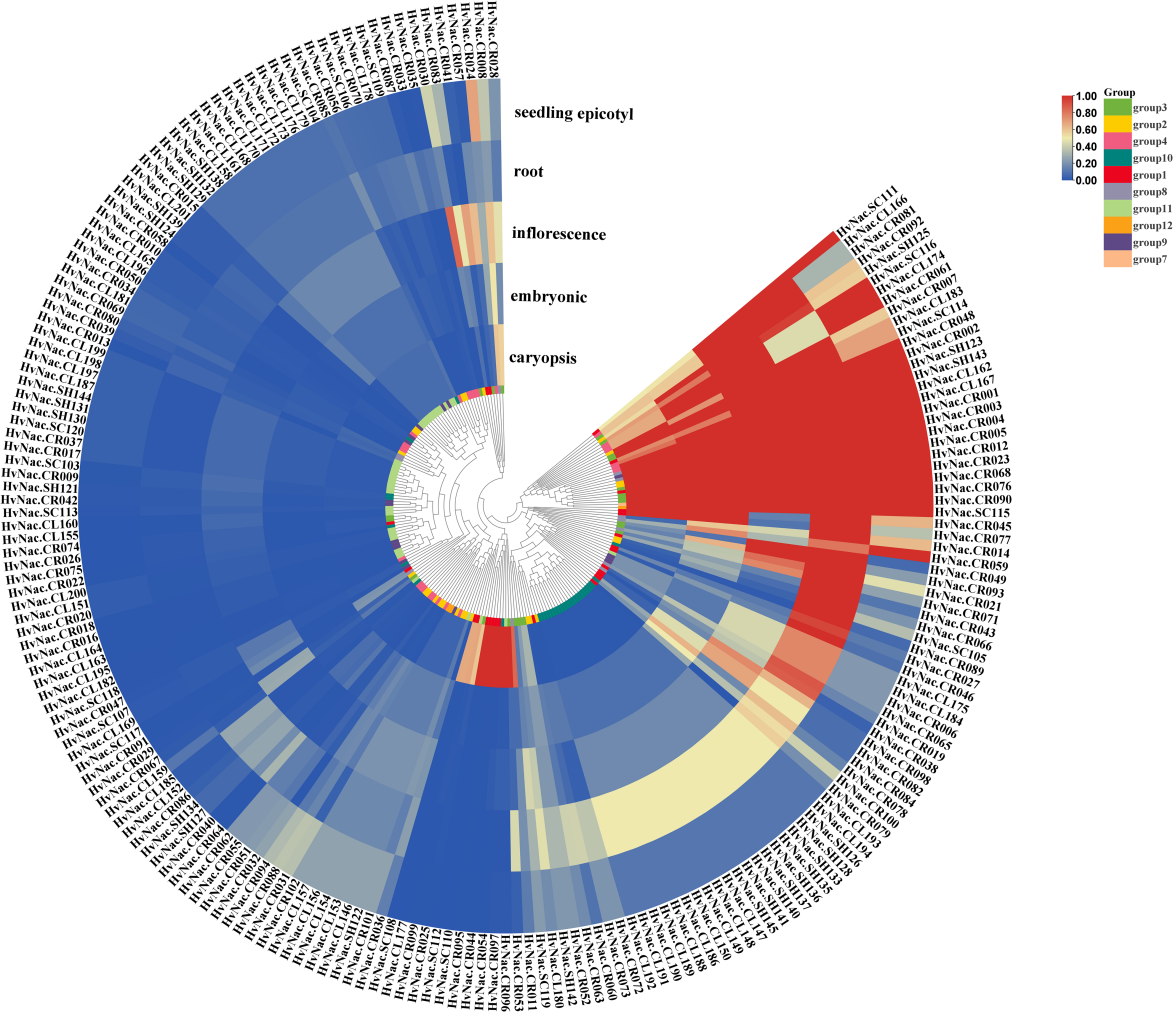
Expression analysis of NAC genes in different barley tissues, based on 20 barley accessions covering five tissue types (caryopsis, coleoptile, inflorescence, root, and shoot). Gene expression values represent the average transcript abundance across the 20 genomes; for non-core genes, the average was calculated only from the genome in which the gene was present. The heatmap was generated using min-max normalization to scale expression values to the range [0, 1] for each gene across tissues.

To explore the tissue-specific expression patterns of NAC transcription factors in barley, we analyzed the pan-transcriptomic transcript abundance of 201 *HvNACs* across five representative tissues consisting of caryopsis, embryonic, inflorescence, root, and seedling epicotyl. These genes were categorized into 12 groups based on their transcriptional signatures among all tissues. The results revealed a pronounced variation in expression profiles among different tissues (**Figure 7**). Notably, Group 7 (containing only one gene) displayed the highest average expression across all tissues, particularly in inflorescence (mean FPKM=91.34) and seedling epicotyl (mean FPKM=77.37). Group 3 and Group 8 also exhibited high expression levels across multiple tissues, with Group 3 showing high expression in roots (mean FPKM = 46.95). Byn contrast, Group 10 and Group 11 were characterized by low to moderate expression across all tissues. Group 10 genes showed relatively higher expression in roots (4.07) but remained low in other organs. Group 11, although widely distributed in the phylogenetic tree, showed consistently low expression levels (mean FPKM<1.0 in all tissues). Group 2 and Group 4 demonstrated moderate and constitutive expression across all tissues, indicating a more conserved and possibly fundamental role in general plant development. Group 1 genes, despite having a wide range of expression levels (standard deviation of 16.84–32.72), showed high expression in roots (mean FPKM =15.16) and caryopsis (mean FPKM =11.53). Overall, the transcriptomic landscape of *HvNACs* reveals both conserved and divergent regulatory roles in barley growth and development.

### Mfuzz clustering analysis of *HvNACs* under salt stress

**Figure 8:**
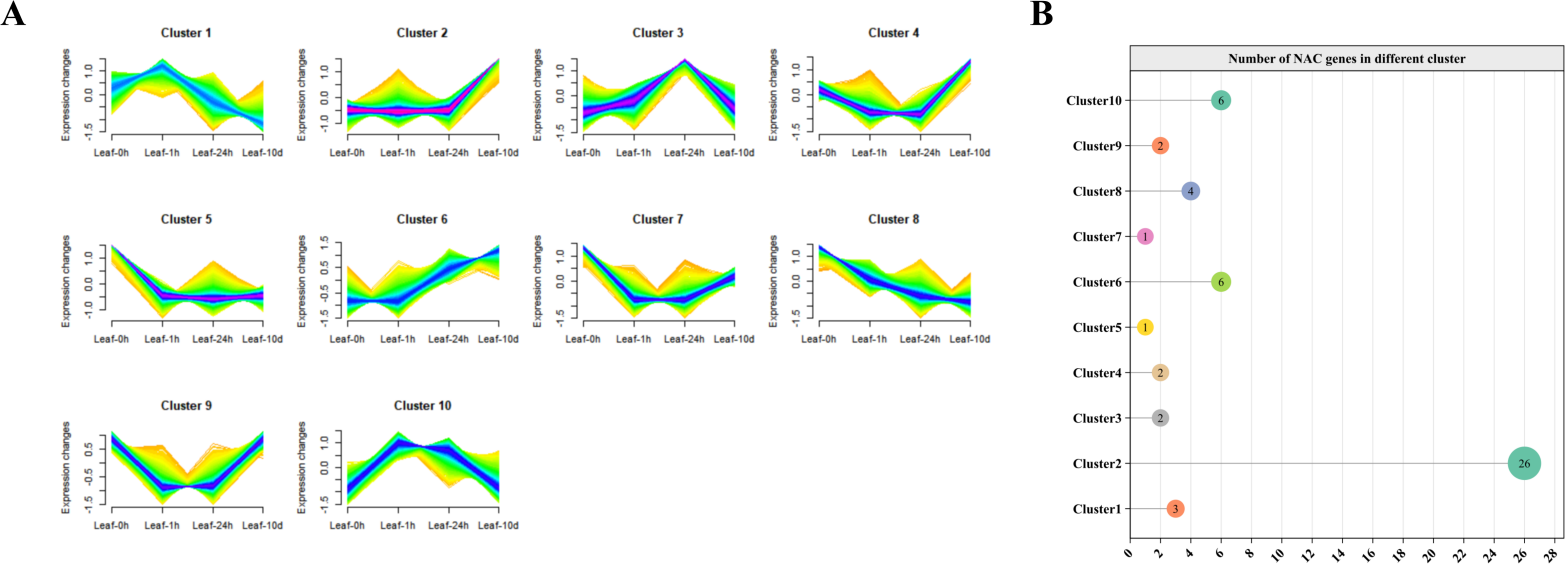
Temporal expression patterns and clustering of HvNAC genes in barley leaves under salt stress. **(A)** Mfuzz clustering analysis in barley leaves under salt treatment at 0h, 1h, 24h, and 10d, with genes showing expression levels greater than 10 into 10 clusters. **(B)** The number of *HvNACs* in each cluster.

To characterize the dynamic transcriptional response of *HvNACs* to salt stress, we analyzed transcriptome data at four time points (0h, 1h, 24h, 10d) after salt treatment. Low-expression genes (average FPKM < 10) were filtered out to enhance analytical robustness. The remaining genes were then subjected to time-series clustering using the Mfuzz algorithm, which grouped them into 10 distinct clusters based on their expression profiles (**Figure 8A**). A total of 53 *HvNACs* were unevenly distributed across these 10 clusters. Cluster 2 contained the largest number (26 *HvNACs*), while Clusters 5 and 7 each included only one (**Figure 8B**). Further analysis of expression trends highlighted diverse regulatory roles of *HvNACs*. Notably, two contrasting profiles emerged: Cluster 6 showed gradual upregulation from 0h to 10d, whereas Cluster 8 exhibited progressive downregulation over the same period. These findings suggest that Cluster 2 (due to its high *HvNAC* enrichment), Cluster 6 (gradual upregulation), and Cluster 8 (progressive downregulation) represent key regulatory modules warranting further investigation into the molecular mechanisms responsible for salt stress response in barley.

**Figure 9:**
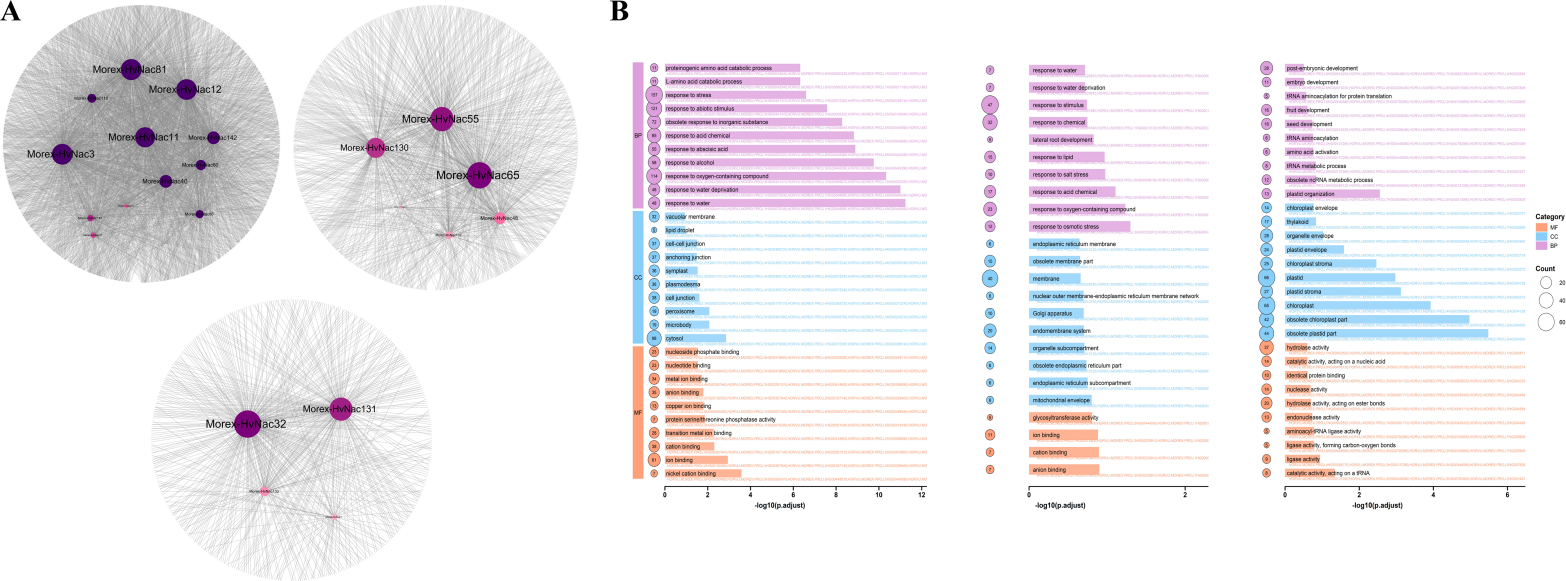
Gene co-expression network and functional enrichment analysis of salt-responsive *HvNACs* clusters. **(A)** Network diagram of genes in clusters 2, 6, and 8 that are highly correlated with *HvNACs* and contain NAC binding sites in their promoter regions. **(B)** GO enrichment analysis of genes in clusters 2, 6, and 8.

Using Pearson correlation analysis, we identified genes highly correlated with *HvNACs* (correlation coefficient > 0.9, p-value < 0.01) within specific clusters under salt stress in barley (**Supplementary Table S11**). The promoter regions of these genes were analyzed for NAC binding sites using FIMO, employing 21 *HvNACs* binding motifs retrieved from the PlantTFDB database (**Supplementary Table S12**). The results revealed 1,075 genes in Cluster 2, 244 genes in Cluster 6, and 362 genes in Cluster 8 with predicted NAC binding sites and high correlation with *HvNACs* (**Figure 9A**), indicating potential transcriptional regulatory networks associated with salt stress response.

GO enrichment analysis of the three key clusters uncovered significant biological processes related to salt stress (**Figure 9B**). In cluster 2, enriched GO terms such as signaling receptor activity (GO:0038023), transferase activity (GO:0016740), carbohydrate homeostasis (GO:0033500), and beta-glucan biosynthesis (GO:0051274) indicate involvement in salt stress signal transduction, carbohydrate metabolism, and cell wall integrity maintenance, with additional roles in abscisic acid signaling and responses to osmotic stress, salinity, and dehydration. Cluster 6 showed enrichment in ion binding (GO:0043167), cation binding (GO:0043169), and response to salt (GO:0009651), highlighting functions in ion transport, homeostasis, and stress adaptation. Conversely, a downregulated cluster 8 exhibited significant enrichment in catalytic activity (GO:0140101), acting on a tRNA (GO:0140101), ligase activity (GO:0016874), and chloroplast (GO:0009507), suggesting reduced RNA processing and photosynthesis-related activities to conserve energy and minimize oxidative damage. Enriched biological processes like response to salt stress (GO:0009651) and carboxylic acid transport (GO:0046942) in this cluster 8 may imply a strategic suppression of non-essential pathways to prioritize stress response resilience, enhancing adaptation to salinity in barley.

## Discussion

The NAC transcription factor family member is a key regulator of plant development and abiotic stress responses across diverse species (Shao et al., 2015). Understanding the evolutionary dynamics and functional diversification of NAC genes in cereal crops like barley is critical for developing stress-resilient varieties through targeted breeding (Fuertes-Aguilar and Matilla, 2024). Our systematic investigation of *HvNACs* at pan-genome level gives novel insights into their evolutionary trajectories and functional specialization patterns in barley.

### Pangenome analysis reveals significant diversity and impact of genome assembly quality

Through systematic analysis of NAC transcription factors in 20 barley pangenome datasets, we obtained critical insights into their genomic architecture, evolutionary patterns, and functional characteristics. A total of 127–149 *HvNACs* were identified in each barley genome, with the majority of accessions containing 130–135 functional members. These variations reflect the natural genetic heterogeneity characteristic of barley pangenome systems, highlighting the rich genetic diversity available for breeding programs aimed at improving specific traits. Compared with other species, the number of NAC genes in barley is higher than in *A. thaliana* (105 genes) (Ooka et al., 2003), comparable to rice (151 genes) (Nuruzzaman et al., 2010); and foxtail millet (147 genes) (Puranik et al., 2013), but lower than in wheat (559 genes) (Guerin et al., 2019). Significant variations in *HvNACs* characteristics (sequence length, molecular weight, isoelectric point, instability index) may indicate extensive functional diversity and adaptive regulatory capacity under environmental stresses (Puranik et al., 2012). The Morex V3 assembly showed maximum NAC gene representation (149), highlighting the critical influence of assembly quality on accurate gene prediction. This conclusion was further corroborated by comparative analysis between Morex-v3 and its predecessor Morex-v2. The increased NAC gene complement in Morex-v3 probably stems from improved assembly continuity, resolution of fragmented genomic regions, and enhanced novel gene annotation. These advancements enabled detection of NAC genes that were either fragmented or undetected in previous assemblies.

### Evolutionary stratification into core and dispensable gene sets

The 201 NAC OGGs were systematically categorized into four evolutionary classes: core genes (present in all accessions), soft-core (≥90% frequency), shell (5 present in >10% but <90% of the accessions), and lineage-specific/cloud genes (≤10% prevalence), establishing a comprehensive evolutionary classification system. Collectively representing 81.2% of *HvNACs*, core and soft-core genes were primarily localized to phylogenetic group 1–4 and clade 8, suggesting their fundamental role in maintaining conserved regulatory networks in barley. The strong conservation of these clades, evidenced by lower Ka/Ks values suggestive of purifying selection, confirms their essential involvement in core cellular processes including developmental regulation and stress response pathways (Panchy et al., 2016). In contrast, shell (25 OGGs) and lineage-specific/cloud genes (56 OGGs) constitute an adaptive reservoir characterized by incomplete pan-genome representation and relaxed selection constraints. This gene cohort likely facilitates environmental adaptation and niche-specific stress tolerance, representing promising targets for future functional studies and breeding efforts to enhance stress resilience. These dispensable genes may represent a valuable resource for identifying novel alleles associated with adaptation to specific local environments or emerging stresses, offering promising avenues for targeted breeding strategies to enhance resilience in diverse agricultural settings (Dawson et al., 2015). Comparative phylogenetics revealed lineage-specific divergence, with group 5 and 6 uniquely conserved in rice NACs. These evolutionary characteristics may imply Poaceae-specific functional specialization, possibly driven by differential domestication histories or ecological adaptation requirements (Doebley et al., 2006).

### Genomic rearrangements and transposable elements drive *HvNACs* dynamic expansion

CNVs and PAVs detected in *HvNACs* demonstrate their evolutionary plasticity. This variability spans conserved single-copy core genes to genotype-specific expansions, exemplified by *HvNAC.CR057* and *HvNAC.CL162/164* amplifications in Morex, may implying dosage-dependent regulation of NAC-mediated pathways. PAVs involving segmental insertions/deletions indicate ongoing genomic restructuring, a phenomenon also reported in wheat NAC genes (Singh et al., 2021).This CNV and PAV diversity may be partly driven by differential selection pressures associated with barley’s adaptation to diverse agro-ecological environments during domestication and improvement (Pourkheirandish and Komatsuda, 2007; Russell et al., 2016). As barley has been cultivated across a wide geographic range—from arid regions in the Fertile Crescent to temperate zones in Europe and East Asia—local adaptation likely favored the expansion or retention of lineage-specific NAC genes with stress-related functions, particularly those associated with drought, salinity, and cold tolerance (Dawson et al., 2015). Additionally, repeated selection bottlenecks and introgressions during modern breeding may have contributed to gene copy variation in elite cultivars.

A strong association emerged between NAC gene diversification and TE mobilization. DNA transposons (85.25% of TE content) predominantly occupied NAC gene flanking regions, with significant enrichment in 5’/3’ regulatory sequences. Spatial correlation between TEs and CNVs suggests TE-mediated local duplication mechanisms, consistent with Poaceae genome evolution patterns (Bennetzen and Wang, 2014; Wicker et al., 2018). In this study, retrotransposons exhibited lower abundance, while retrotransposons are responsible for increasing genomic flexibility as revealed previously (Chen et al., 2023). Collectively, these data suggest that TEs act as primary architects of NAC gene diversity, which likely contributes to barley adaptation coping with environmental stresses. The observation that dispersed duplication is the dominant mechanism (56.37%) for NAC expansion, coupled with enrichment at recombination-prone distal chromosome ends, further supporting the role of TEs and genome instability in shaping the molecular evolution of *HvNACs*. Understanding the interplay between TEs and NAC gene evolution provides critical insights into the mechanisms driving adaptive evolution in barley, potentially enabling future strategies for manipulating TE activity to generate beneficial genetic variations for crop improvement.

### Functional insights from structure, codon usage, and expression patterns

The consistent exon-intron structures and conserved motifs within NAC subfamilies suggest a common evolutionary origin. However, subtle differences among clades, such as the absence of Motif_1 in Group 10, the presence of Motif_7 and Motif_8 in Group 11, and the emergence of unique domains in lineage-specific genes, may reflect functional specialization and adaptive evolution of *HvNACs*.

Codon usage analysis revealed a generally weak codon bias among *HvNACs*, indicating that the translation efficiency of the most genes is not likely to undergo strong selective pressure. The identified preferred codons provide valuable clues for optimizing gene expression in future genetic engineering applications. At the same time, the neutral deviations observed in the ENC and PR2 plots, may implying both mutational pressures, especially GC bias, and possible translational selection contribute to codon preference. These factors may modulate expression efficiency or influence protein folding of certain genes under specific conditions. Furthermore, a set of preferred and optimal codons, predominantly ending in G or C, was identified. As this analysis was based on 20 barley genomes, the limited sample size may constrain the generalizability of the codon usage patterns observed. A larger genomic dataset could enhance the robustness of these findings. This analysis provides preliminary insights into the evolutionary forces influencing codon preferences in *HvNACs*, with potential applications for gene expression optimization.

Tissue expression profiling revealed that *HvNACs* show pronounced expression differences across various tissues, and these expression patterns are closely associated with their phylogenetic classifications. Genes belonging to Group 3 and Group 8 showed high expression levels in roots and inflorescences, suggesting their roles in tissue-specific developmental processes. This pattern is consistent with the reported functions of NACs in tissue differentiation and secondary cell wall formation (Olsen et al., 2005; Zhong et al., 2010). Genes in Group 2 and Group 4 exhibited relatively stable expression patterns, suggesting potential housekeeping roles. In contrast, the low expression levels of Group 10 and Group 11 suggest possible involvement in developmental stages or stress conditions not examined in this study, or in regulatory functions that require minimal transcriptional activity. Moreover, the considerable variation in expression within Group 1 highlights the functional diversity in *HvNACs*.

### *HvNACs* involve in salt stress response

Using Mfuzz clustering analysis, we identified the dynamic transcriptional responses of *HvNACs* to salt stress, supporting their essential roles in barley adaptation coping with abiotic stress. Interestingly, among all clusters, only cluster 2 and cluster 8 each contained a single gene classified as soft-core, while the remaining *HvNACs* in these stress-responsive clusters were core genes. This pattern indicates that the transcriptional response to salt stress is primarily mediated by evolutionarily conserved NAC genes, and that core NACs may play dominant roles in the regulation of key stress adaptation pathways. The presence of a few SC genes may reflect lineage-specific modulation or fine-tuning of stress responses in certain accessions.

Cluster 2 (*HvNACs* high-enriched) and Cluster 6 (upregulated expression after salt stress) are associated with signal transduction, ion homeostasis, and cell wall integrity, all of which are crucial for salt tolerance (Zhu, 2002). Within Cluster 6, we identified two *HvNACs*, *Morex-HvNac55* and *Morex-HvNac65*, which show high co-expression with multiple salt stress-responsive genes, including regulate signal transduction, ion homeostasis, and cell wall integrity, crucial for salt tolerance. The downregulation of Cluster 8 indicates a possible inhibitory role, potentially preventing excessive activation through modulation of stress responses (Hu et al., 2008). GO enrichment analysis of these clusters revealed that *HvNACs* are involved in several key stress response pathways, including signal perception and transduction (e.g., receptor activity, ABA signaling), metabolic regulation (carbohydrate homeostasis), ion homeostasis (ion/cation binding), and cellular protective functions (response to stimuli, dehydration, osmotic stress). This finding is consistent with previous studies emphasizing the role of NAC transcription factors in regulating abiotic stress tolerance in various plant species (Nakashima et al., 2009; Tran et al., 2010; Shao et al., 2015). These key *HvNACs*, especially *Morex-HvNac55* and *Morex-HvNac65*, are strong candidates for molecular breeding and transgenic strategies aimed at enhancing salt tolerance in barley.

## Conclusion

This study provides the comprehensive pangenome-scale characterization of the NAC transcription factor family in barley. By integrating analyses of gene content, phylogeny, structural variation, selection pressure, codon usage, and expression, we have unveiled the complex evolutionary history and functional diversification of *HvNACs*. Our findings indicate that a ranging from 127 to 149 *HvNACs* are identified across 20 barley accessions, reveal distinct evolutionary trajectories for core versus dispensable genes in *HvNACs*, underscore the pivotal role of TEs and genomic rearrangements in driving the expansion of *HvNACs*, and provide strong evidence for the involvement of specific NAC subsets in tissue development and salt stress responses. These findings establish a valuable foundation for future functional genomics studies and may inform efforts to explore the roles of specific *HvNACs* in stress response and developmental pathways. In addition, this work could support barley improvement programs by identifying candidate genes for enhancing stress tolerance and agronomic performance. Future research directions may include functional validation of key *HvNACs* using gene editing and transcriptomic approaches, as well as the incorporation of *HvNACs* diversity into marker-assisted selection to facilitate the development of barley varieties adapted to a range of agro-ecological conditions.

## Data Availability

The original contributions presented in the study are included in the article/**Supplementary Material**. Further inquiries can be directed to the corresponding author.
