# Supplementary figures and images for: The evolution, variation, and expression patterns under development and stress responses of the NAC gene family in the barley pan-genome
Source: Front Plant Sci. 2025 Aug 7;16:1635416. doi: 10.3389/fpls.2025.1635416 (PMC12367700; doi:10.3389/fpls.2025.1635416)

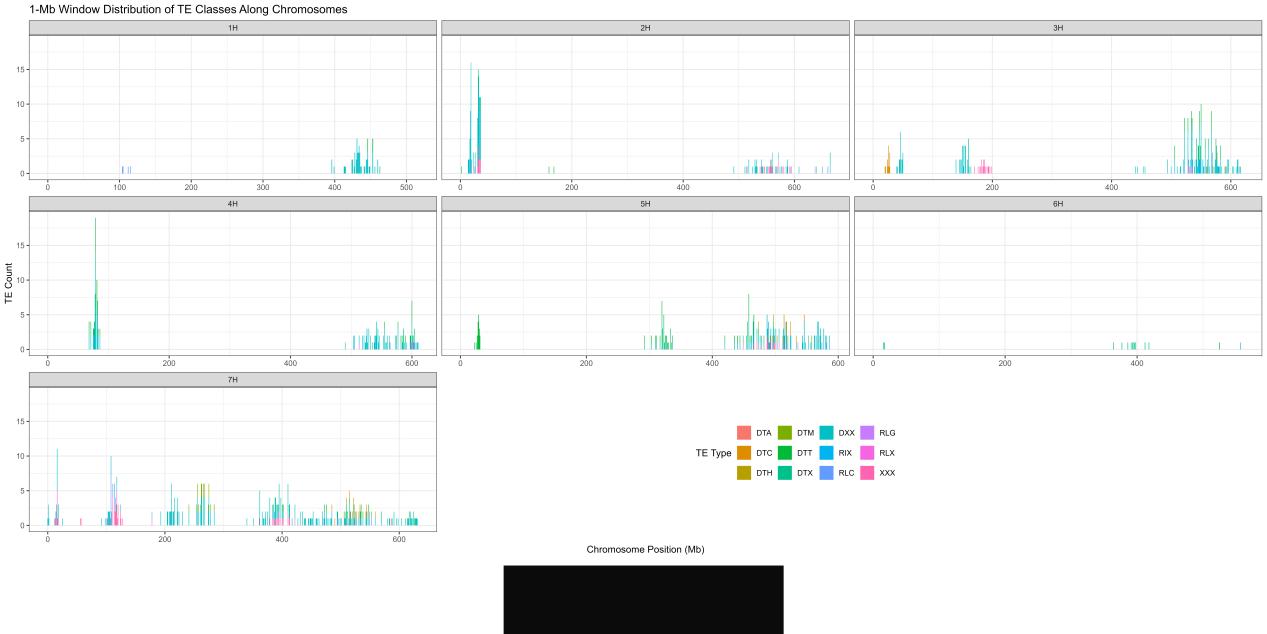


**Figure S1.** Distribution of different TE types along chromosomes calculated in 1 Mb windows.

Supplement: Supplementary file 2 [file Table2.docx]
